# Brachial Plexus Injury Influences Efferent Transmission on More than Just the Symptomatic Side, as Verified with Clinical Neurophysiology Methods Using Magnetic and Electrical Stimulation

**DOI:** 10.3390/biomedicines12071401

**Published:** 2024-06-24

**Authors:** Agnieszka Wiertel-Krawczuk, Agnieszka Szymankiewicz-Szukała, Juliusz Huber

**Affiliations:** Department Pathophysiology of Locomotor Organs, Poznań University of Medical Sciences, 28 Czerwca 1956 Str. No 135/147, 61-545 Poznań, Poland; wiertelkrawczuk@ump.edu.pl (A.W.-K.); aszymankiewicz@ump.edu.pl (A.S.-S.)

**Keywords:** brachial plexus injury, cervical roots, Erb’s point, magnetic stimulation, electrical stimulation, electroneurography, electromyography

## Abstract

The variety of sources of brachial plexus injuries (BPIs) and the severity and similarity of their clinical symptoms with those of other injuries make their differential diagnosis difficult. Enriching their diagnosis with objective high-sensitivity diagnostics such as clinical neurophysiology may lead to satisfactory treatment results, and magnetic stimulation (MEP) might be an advantageous addition to the diagnostic standard of electrical stimulation used in electroneurography (ENG). The asymptomatic side in BPI cases sometimes shows only subclinical neurological deficits; this study aimed to clarify the validity and utility of using MEP vs. ENG to detect neural conduction abnormalities. Twenty patients with a BPI and twenty healthy volunteers with matching demographic and anthropometric characteristics were stimulated at their Erb’s point in order to record the potentials evoked using magnetic and electrical stimuli to evaluate their peripheral motor neural transmission in their axillar, musculocutaneous, radial, and ulnar nerves. MEP was also used to verify the neural transmission in participants’ cervical roots following transvertebral stimulations, checking the compatibility and repeatability of the evoked potential recordings. The clinical assessment resulted in an average muscle strength of 3–1 (with a mean of 2.2), analgesia that mainly manifested in the C5–C7 spinal dermatomes, and a pain evaluation of 6–4 (mean of 5.4) on the symptomatic side using the Visual Analog Scale, with no pathological symptoms on the contralateral side. A comparison of the recorded potentials evoked with magnetic versus electrical stimuli revealed that the MEP amplitudes were usually higher, at *p* = 0.04–0.03, in most of the healthy volunteers’ recorded muscles than in those of the group of BPI patients, whose recordings showed that their CMAP and MEP amplitude values were lower on their more symptomatic than asymptomatic sides, at *p* = 0.04–0.009. In recordings following musculocutaneous and radial nerve electrical stimulation and ulnar nerve magnetic stimulation at Erb’s point, the values of the latencies were also longer on the patient’s asymptomatic side compared to those in the control group. The above outcomes prove the mixed axonal and demyelination natures of brachial plexus injuries. They indicate that different types of traumatic BPIs also involve the clinically asymptomatic side. Cases with predominantly median nerve lesions were detected in sensory nerve conduction studies (SNCSs). In 16 patients, electromyography revealed neurogenic damage to the deltoid and biceps muscles, with an active denervation process at work. The predominance of C5 and C6 brachial plexus injuries in the cervical root and upper/middle trunk of patients with BPI has been confirmed. A probable explanation for the bilateral symptoms of dysfunction detected via clinical neurophysiology methods in the examined BPI patients, who showed primarily unilateral damage, maybe the reaction of their internal neural spinal center’s organization. Even when subclinical, this may explain the poor BPI treatment outcomes that sometimes occur following long-term physical therapy or surgical treatment.

## 1. Introduction

Overload forces are the leading cause of brachial plexus injuries (BPIs), causing damage to the shoulder girdle, muscle tissue, and blood vessels. The complexity of a BPI is often accompanied by severe multi-organ injuries, their associated diagnostic difficulties, and the choice of an optimal treatment [1,2]. Moreover, diagnostics after a BPI to evaluate the sensory and motor function of the arm and distal part of the upper extremity sometimes need to be postponed due to the patient’s general poor health status, negatively influencing their treatment and prognosis [3,4]. The absence of a “gold standard” for the assessment of the severity of BPI lesions complicates prognostic studies of BPI [5]. The routine examination of the consequences of brachial plexus damage, as with other peripheral neuropathies, is mainly based on clinical tests [6]. This examination primarily includes a functional assessment of the shoulder girdle’s muscle strength, the range of the patient’s structural atrophic changes, their sensory perception deficits, and their range of motion both in the shoulder joint and in other joints of the upper extremity [7]. Both magnetic resonance imaging and ultrasound examinations enhance clinical diagnoses by analyzing the location and extent of the structural damage within injured nerves and surrounding tissues [8,9]. The variety of sources of brachial plexus injuries, the degree of their scope and severity, and the similarity of their clinical symptoms make it difficult to choose between surgery and continuing physiotherapy [10]. A differential diagnosis should be based on modern objective methods with high sensitivity; currently, clinical neurophysiology tools are preferred [11]. Neuroimaging and neurophysiological tests should be an integral part of the diagnostic process, guiding the selection of surgical or conservative treatments. Optimal diagnostic results are obtained following a comparison of the neuroimaging and functional evaluation of injured neural structures, as is the case for patients after an incomplete spinal cord injury [12]; however, this opinion has not yet been approved in cases of patients with BPI. In studies by Chanlalit et al. [13], the diagnostic value of their clinical findings, electromyography, and magnetic resonance imaging of root lesions in traumatic brachial plexus injuries was evaluated to be 60, 87, and 70%, respectively. The results of clinical neurophysiology tests of the neural conduction of motor and sensory fibers are analyzed following electroneurographic (ENG) recordings. The stimulus used to excite nerve fibers and generate motor potentials (CMAPs—compound muscle action potentials, sometimes identified as M-waves) and sensory potentials (SNAPs—sensory nerve action potentials) is an electrical pulse of known intensity and duration. The use of ENG tests in the diagnosis of nerve-damaged peripheral parts is widely described in the literature and also applied to the functional evaluation of BPIs [14]. In our previous work [15,16], we extensively studied and discussed the usefulness of a magnetic stimulus (MEP—motor-evoked potential) in the evaluation of the function of brachial plexus motor fibers. Magnetic stimulation is an advantageous addition to the diagnostic standard used in BPI cases. It allows for evaluating the function of the cervical roots, which are the origin of the peripheral innervation of the upper extremities’ muscles. MEPs are crucial for the functional confirmation of the avulsion range and compression of cervical spinal roots. Magnetic cervical motor root stimulation is useful for detecting abnormal findings in the upper part of the brachial plexus, even at the acute phase of a BPI [17]. The compatibility of imaging studies and neurophysiological tests in evaluating the proximal part of the brachial plexus is essential, especially for reconstructive surgery. Moreover, extended neurophysiological diagnostics (ENG, MEP) may also include the asymptomatic side of the injury, which sometimes shows subclinical neurological deficits in basic examinations that are often ignored or neglected. Our research is driven by insufficient scientific data in the field of neurophysiological BPI diagnostics regarding the application of complementary MEP and ENG tests. Combining both examination techniques allows us to assess the entire neuromere, which is of particular diagnostic importance in proximal BP lesions located at the level of the spinal roots or spinal nerves. So far, the diagnostic gap in the function of the brachial plexus has been caused by the lack of direct assessments of its proximal parts. MEP tests allow us to assess the function of the spinal roots, which complements the neurophysiological diagnostics performed on BPI patients. In combination with an imaging examination (MRI), this fulfills the complementary diagnosis of BPI. In this study, we answer the question of whether the use of stimulation with physically different stimuli (electrical vs. magnetic) allows for an objective and repeatable assessment of BPI patients. We also attempted to verify our hypothesis, which is that both types of stimuli can be used interchangeably for diagnostic purposes. Our next question concerns the validity of extended neurophysiological diagnostics in assessing the level, range, and type of brachial plexus damage present and whether functional changes are also present on the clinically asymptomatic side. We additionally hypothesized that an injury to one side of the brachial plexus may also cause pathological symptoms in contralateral neural structures, either as a consequence of the spinal reaction to the structural shock or due to adaptative changes, which are the reaction of the internal spinal motor centers. In this report, the “asymptomatic side” is considered to present no clearly clinically detected sensory or motor impairments, which does not mean that abnormalities cannot be observed in its neurophysiological recordings. These are the main aims of the presented study.

## 2. Materials and Methods

### 2.1. Participants, Study Design, and Clinical Evaluation

The results of single neurophysiological recordings performed in 20 patients with brachial plexus injury and 20 healthy volunteers were analyzed in this study (Table 1).

Subjects were examined in the Department of Pathophysiology of Locomotor Organs in the Wiktor Dega Orthopedic and Rehabilitation Hospital of Poznań University of Medical Sciences. The inclusion criterion for the patients in the research group was a diagnosis of brachial plexus injury (BPI) based on clinical examinations, including manual muscle strength testing, sensory perception evaluations with reference to the spinal dermatomes, pain evaluations using the visual analog scale, and magnetic resonance imaging (MRI) of their symptomatic and asymptomatic upper extremities. Manual muscle strength testing was performed on the proximal and distal muscle groups in the upper extremities using a Lovett scale (0–5). It consists of six grades that assess different levels of muscle strength (0—no visible voluntary contraction of the muscle, 5—normal muscle strength) [18,19]. Their sensory perception was assessed (0—analgesia, 1—normal, 2—hyperesthesia) according to the dermatomal scheme for the innervation of the brachial plexus sensory fibers, which is based on a tactile method using Von Frey’s filaments (Semmes–Weinstein monofilaments) [20,21]. Pain intensity was evaluated using a patient-reported 10-point visual analog scale (VAS) [22]. The time from the onset of the BPI to the clinical and neurophysiological evaluation of the patients was 4 months on average.

The exclusion criteria and contraindications for MEP examinations in the patients and healthy volunteers included pregnancy, epilepsy, cardiac disease, and the use of a pacemaker or other implanted biomedical electronic devices [23]. Moreover, for the control group of healthy volunteers, the exclusion criteria also included neck, head, and shoulder girdle injuries.

Our ethical considerations adhered to the Helsinki Declaration. Approval was received from the Bioethical Committee of the University of Medical Sciences in Poznań, Poland (including our studies on healthy people), Decision No. 554/17. Each participant signed a written consent form for their examination and the publication of their data; they were informed about the project’s aims. The patients were routinely diagnosed in the hospital’s clinical neurophysiology unit. A physician performed clinical studies and described the results in the patients’ medical history. Three experienced clinical neurophysiologists conducted the MEP, ENG, and electromyography (EMG) studies. All of them judged the final results after the consultations. The patients’ medical histories were analyzed to determine the causes of their brachial plexus injury.

The group of patients was older than the healthy volunteers (Table 1), and the difference was close to the level of statistical significance. All other differences in the anthropometric properties of the studied subjects were statistically non-significant, and the age of the healthy volunteers was within three decades of that of the patients, thus maintaining the possibility of comparing the parameters of their neurophysiological tests. Moreover, the most extreme age values in the research group, i.e., 15 and 66 years old, were limited to single cases. Both groups were homogeneous in terms of height and handedness.

### 2.2. Neurophysiological Examinations

The patients with BPI and the healthy volunteers were examined bilaterally using the same neurophysiological scheme. We used magnetic and electrical stimuli to evaluate the peripheral motor neural transmission in the four nerves that create the brachial plexus: the axillar, musculocutaneous, radial, and ulnar nerves. The magnetic stimulus was also used to verify neural transmission in the cervical roots according to the domain of the above-mentioned peripheral nerves and muscles (Table 2). We stimulated these nerves at Erb’s point and in specific parts of the cervical segment several times, checking the repeatability of the evoked potentials. All studies were performed in the same quiet diagnostic room with constant, pleasant humidity and a temperature of 22 °C. During the ENG examination, the subjects were in a supine position; during the MEP recordings, they were in a sitting position, with the muscles of their upper extremities relaxed and their shoulders freely positioned.

The multichannel KeyPoint Diagnostic System (Medtronic A/S, Skøvlunde, Denmark) was used for the MEP and CMAP (ENG, also called M-waves) recordings. The external magnetic stimulus for the MEP studies was generated by the MagPro X100 magnetic stimulator (Medtronic A/S, Skøvlunde, Denmark) via a circular coil (C-100, 12 cm in diameter) and applied to Erb’s point or transvertebrally at different cervical spine levels. During the MEP studies, the strength of the magnetic field stream was 100% of the maximum stimulus output (1.7 T for each pulse). The recordings were performed at an amplification of 20 mV/D and a time base of 5–8 ms/D. For the CMAP recording, a bipolar stimulation electrode and a single rectangular electric stimulus were used at a 1 Hz frequency for 0.2 ms. The intensity of the electric stimulus was about 100 mA, which evoked the supramaximal CMAP amplitude at Erb’s point. The strength of the electrical stimulus was recommended and determined by anatomical relationships and exposure of the neural structures of the brachial plexus at the level of the supraclavicular fossa. In the ENG studies, the time base was set to 5 ms/D, the sensitivity of the recordings was set to 2 mV/D, and upper and lower filters of 10 Hz and 10 kHz were established on the recorder amplifier. A bipolar stimulation electrode was used, and pools were moisturized with a saline solution (0.9% NaCl). The skin was disinfected with a 70% alcohol solution. This and a conductive gel reduced the resistance between the skin and the recording electrodes. The impedance did not exceed 5 kΩ. In the ENG study, bipolar stimulating electrodes were applied at Erb’s point, with the orientation of the stimulus’s delivery in the orthodromic direction for the excitation of the motor fibers in the nerves. The electrical stimulation generated the CMAP recording with the shortest latency value and the highest amplitude, which became the aim of magnetic stimulation at this level. The assessment of the MEP evoked in the spinal roots of the cervical segment required the magnetic coil to be placed 0.5 cm laterally and selectively below the spinous process (in pursuance of the anatomical location of the cervical spinal roots).

For the recordings of the CMAPs and MEPs, standard disposable Ag/AgCl electrodes with an active surface area of 5 mm^2^ were used and placed in the same location when electric or magnetic stimuli were applied. The active electrode was placed over the belly muscle, and the reference electrode was placed distally to the active one, on the olecranon or the muscle tendon [24]. A list of the tested muscles, their innervation (peripheral pathway and root domain), the location of the recording electrodes, and the type of applied stimuli are presented in Table 2. This table also includes the symptoms of functional loss seen following BPI.

**Table 2:** Summary of the CMAP and MEP methodology (examined muscles, their peripheral innervation, and root domain) according to Ferrante [25], Leis [24], and our previous studies [15] with own modifications, as well as the symptoms of functional loss seen according to Park et al. [6].

Muscle (CMAPs and MEPs Recordings)	Electrical and Magnetic Stimulation of Nerves at Erb’s Point	Brachial Plexus Trunk	Cervical Root (Magnetic Stimulation at a Significant Root Domain)	Function Loss
Deltoid (Middle part) Active electrode—belly muscle Reference electrode—olecranon	Axillary nerve	Upper	C5–C6 (C5)	Shoulder rotation and abduction
Biceps Brachii Active electrode—belly muscle Reference electrode—olecranon	Musculocutaneous nerve	Upper	C5–C6 (C6)	Elbow flexion
Triceps Brachii (Long head) Active electrode—belly muscle Reference electrode—olecranon	Radial nerve	Upper Middle Lower	C6–C8 (C7)	Elbow extension
Abductor Digiti Minimi Active electrode—belly muscle Reference electrode—muscle tendon	Ulnar nerve	Lower	C8–T1 (C8)	Ulnar intrinsic muscle stretching (ADM)

Abbreviations: CMAP—compound muscle action potential; MEP—motor-evoked potential; ADM—abductor Digiti Minimi muscle.

The same output parameters were analyzed for the compound muscle action potential (CMAP) recorded during the electroneurography examination (ENG) and the motor-evoked potential (MEP) induced by magnetic stimulation. The amplitude of the negative deflection (from the baseline to the negative peak—measured in mV) and its latency (from the visible stimulating artifact to the negative deflection of potential—measured in ms) were analyzed.

Confirmation of the axonal type of the brachial plexus injuries (axonotmesis or neurotmesis) required needle electromyography (nEMG) recordings from the muscles of the arm and the distal part of the upper extremity. We analyzed the muscles’ spontaneous activity at rest (denervation potential: fibrillation, positive waves), the parameters of twenty motor unit action potentials (MUAPs), and the frequency of MUAP recruitment during maximal voluntary contraction [26]. Conducting the two last stages of the nEMG recordings was only possible when the voluntary movement of the muscle was available. Two MUAP parameters with values greater than they were at the patients’ referral determined the neurogenic advancement of these patients’ injury and the reinnervation process in their examined muscles. In addition to assessing the function of the motor fibers of the brachial plexus, the ENG examination also examines their sensory component. The same diagnostic system was used for sensory nerve conduction studies (SNCS). Recording electrodes were placed over the skin of the examined nerve passage and along its anatomical course. A ring-type stimulating electrode was placed on a specific finger for the evaluation of the median and ulnar nerves and over the dorsolateral edge of the radius bone during the examination of the radial nerve.

### 2.3. Statistical Analysis

Data analysis was performed using Statistica, version 13.1 (StatSoft, Kraków, Poland). Descriptive statistics are reported as minimal and maximal values (range), with the mean and standard deviation (SD) given, and with the median value given for some clinical test results. The normality distribution and homogeneity of the variances were studied using Shapiro–Wilk and Levene’s tests. None of the collected data had a normal distribution or were of the ordinal scale type. All neurophysiological tests were conducted on a group of healthy volunteers to achieve the normative parameters used to compare the health status of the patients and the controls. In cases where the distribution was not normal, a Mann–Whitney U test was used. Student’s *t*-test (paired difference *t*-test) or Wilcoxon’s test (in the absence of distribution normality) was used to compare the differences between the results obtained for the patients and healthy volunteers. *p*-values of ≤0.05 were considered statistically significant. We also compared the differences with those calculated using the Bonferroni correction at *p* < 0.05. The results did not reveal any significant difference in the parameter values recorded in the neurophysiological tests conducted on the left and right sides of the controls. Attention was paid to matching patients’ and healthy volunteers’ demographic and anthropometric properties, including their gender, age, and height. Statistical software was used to determine the required sample size using the primary outcome variable of the MEP amplitudes recorded in the ADM muscles, with a power of 80% and a significance level of 0.05 (two-tailed) required. The mean and standard deviation (SD) were calculated using the data from the first 10 patients, and the sample size software estimated that more than 15 patients were needed for this study. This population was increased to 20 to provide the most reliable data for statistical analyses.

## 3. Results

Table 3 summarizes the data on the etiology of the brachial plexus damage in the patient group and distinguishes between the types of injuries according to their location.

Brachial plexus injuries related to traffic accidents dominated. These were preganglionic injuries that mainly affected the C5 and C6 spinal roots. Magnetic resonance imaging (MRI) confirmed root avulsion at these levels. The remaining patients’ injuries involved both the roots and trunks of the brachial plexus. Damage to the upper and middle trunks was mainly detected. One patient had iatrogenic damage associated with the surgical removal of neuromas in the anterior triangle of the neck and obstetric injuries. In general, on the symptomatic side of all patients in this study, the clinical neurological assessment made using classical evaluation methods revealed that the muscle strength of the deltoid, biceps, and triceps brachii, as well as the abductor digiti minimi, was 3–1 (mean of 2.2), the sensory perception of the spinal C5–C7 dermatomes were 1 or 0 (median of 0), and the pain experiences were rated as 6–4 (mean of 5.4) using the visual analog scale. No pathological symptoms were detected on the contralateral side using these clinical evaluation methods.

Table 4 presents the results of the neurophysiological studies (ENG, MEP) conducted on patients and healthy volunteers. In the group of healthy volunteers, there was no statistical difference between the left and right sides in terms of the parameters of the amplitudes or latencies of the potentials evoked with electrical stimulation. Following magnetic stimulation at Erb’s point, only in the ADM-ulnar nerve recordings was the amplitude of the MEP higher on the right side than on the left, at *p* = 0.04. A comparison of the recording parameters evoked with magnetic versus electrical stimuli indicated that the amplitudes were higher in the left TB-axillary nerve following electrical compared to magnetic stimulation, at *p* = 0.04. Additionally, the ADM-ulnar nerve recordings on both sides were characterized by amplitudes of MEP higher than those evoked by the electrical stimulation, at *p* = 0.03. In the group of patients, recordings from all examined muscles showed that their CMAP and MEP amplitudes were significantly lower on their symptomatic side, at *p* = 0.04–0.009, compared to the control group.

**Figure 1:**
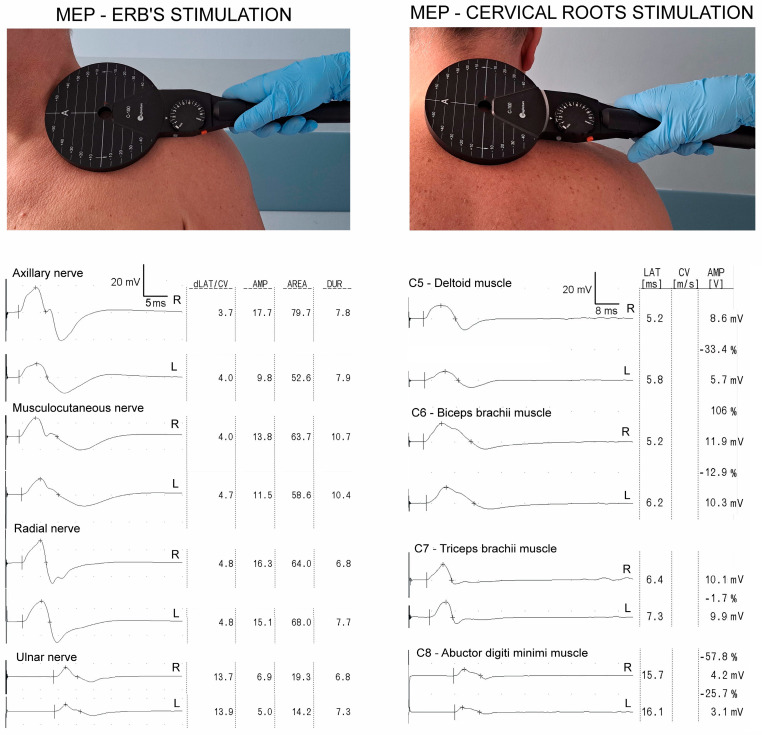
Photographs presenting the placement of the magnetic coil during MEP studies after stimulation at Erb’s point, with transvertebral as well as MEP recordings of both sides (R—right, L—left), respectively, with the amplitude (AMP), latency (dLAT, LAT), and duration (DUR) of the evoked potentials presented.

In the TB-radial and ADM-ulnar nerve recordings obtained following electrical stimulation at Erb’s point and in the ADM-ulnar nerve recording after magnetic stimulation at Erb’s point, the amplitudes of the potentials evoked in the patients were also lower on the asymptomatic side, *p* = 0.04 and *p* = 0.02, respectively, compared to the control group. The MEP amplitudes after the cervical roots were stimulated (C5–C8) were lower in the patients compared to the control group, both on their symptomatic and asymptomatic sides, at *p* = 0.04–0.008. In the BPI patients’ recordings, their latency parameters were longer on their symptomatic side, at *p* = 0.02–0.009, in all but the DP-axillary nerve recordings. During magnetic stimulation at Erb’s point, only in the BB-musculocutaneous nerve recordings on the symptomatic side was the latency longer than that of the asymptomatic side, at *p* = 0.008. Furthermore, in recordings following musculocutaneous and radial nerve electrical stimulation and ulnar nerve magnetic stimulation at Erb’s point, the patients’ latencies were also longer on their asymptomatic side compared to those of the control group. The above outcomes prove the mixed types of brachial plexus injury seen, which are of an axonal and demyelination nature. They lead to the conclusion that a traumatic BPI of mixed types also involves the clinically asymptomatic side. The recordings presented in Figure 1 were obtained from a patient with a superior trunk injury to their brachial plexus after a fall from height. Notice that the amplitude of the CMAP from the axillary nerve was reduced after stimulation of the cervical root C5 on the left side (the symptomatic side of their injury).

In our neurophysiological examinations, evoked potentials with lower amplitudes (CMAP, MEP) indicate an axonal loss in the nerves. This type of nerve injury may refer to axonotmesis or neurotmesis in Seddon’s classification, depending on the severity of the axonal loss. A lack of recording potential implies neurotmesis or severe axonotmesis. The above results indicate predominant axonotmesis in the group of patients with BPI.

**Figure 2:**
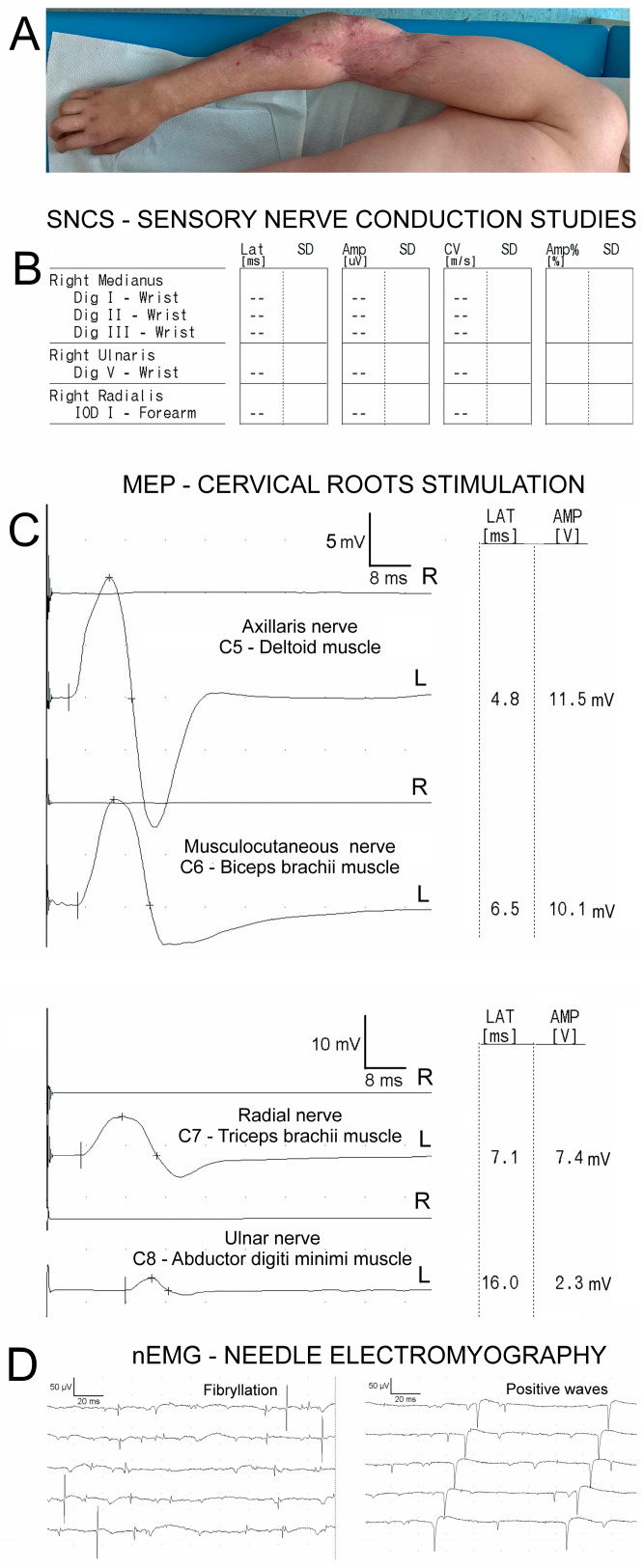
Examples of results from the sensory nerve conduction studies (**B**), MEP recordings after the magnetic stimulation of the cervical roots (**C**), and spontaneous activity during needle electromyography tests (**D**), showing denervation potentials (fibrillation and positive waves). These relevant results were obtained in a patient (**A**) with a total brachial plexus injury and cervical root avulsion on the right side. Abbreviations: N—nerve; Musculocut.—Musculocutaneous nerve; ADM—abductor digiti minimi muscle; C5–C8—cervical root levels.

Our sensory nerve conduction studies (SNCSs) were dominated by cases presenting with median nerve injuries (Table 5). Methodologically, they were performed following the stimulation of receptors within the first finger, which corresponds primarily to the extent of innervation at the level of the brachial plexus in the upper trunk. A neurogenic injury of the deltoid and biceps brachii muscles and an active denervation process were diagnosed in 16 patients through electromyographic recordings (nEMG). The above confirms the predominance of injuries to the C5 and C6 cervical roots and the upper trunk of the brachial plexus in patients. Injuries within the sensory fibers of the other nerves examined, i.e., the radial and ulnar nerves, as well as neurogenic damage to the triceps brachii and dorsal interosseous muscles I occurred in 8 and 7 and 13 and 12 patients, respectively, out of the 20. Therefore, lesions in the middle and lower trunks of the brachial plexus and the cervical roots C7 and C8 occurred less frequently in the group of patients. Patients with damage to their entire brachial plexus, a co-occurring avulsion of their spinal roots, and a lack of evoked potentials and electrical muscle activity were included in the statistical analysis of the neurophysiological test results (Figure 2).

## 4. Discussion

This paper deals with the results of applying MEP vs. ENG diagnostic methodologies to patients with a BPI compared to healthy subjects [16] and records the results found when conducting these neurophysiological evaluations on the symptomatic versus asymptomatic side of patients.

The most important results are the CMAP and MEP amplitude changes and higher latency parameters seen in all examined nerves on patients’ symptomatic side after they were stimulated at their Erb’s point. Importantly, lower amplitudes of CMAP in the axillary and ulnar nerves and MEP in the ulnar nerve were recorded on the patients’ asymptomatic side compared to those of the control group. Recordings following musculocutaneous and radial nerve electrical stimulation and ulnar nerve magnetic stimulation at Erb’s point showed that the patients’ latencies were also longer on their asymptomatic side compared to those of the control group. The sensory deficits seen in the SNCSs were dominated by cases with predominantly median nerve lesions, similar to Jones et al.’s study [27]. Interestingly, the MEP amplitudes from the recordings of all evaluated C5–C8 cervical root levels in patients were reduced bilaterally compared to the recordings from healthy volunteers. The above results indicate that the asymptomatic side of these BPI patients also demonstrates symptoms of lesions. Contemporary data may explain this phenomenon as being due to the high overloading forces that were the mechanism of injury, which were not only unilateral [28]. They may cause the direct stretch or rupture of nerves, cervical roots, or even spinal cord tissues, leading to associated edema or arterial blood flow abnormalities [29,30]. The second explanation could be related to the adaptative processes of the intraspinal neuronal connections engaged in sensory perception and movement coordination, but this theory has only been studied under experimental conditions so far [31]. Both phenomena are related to the small distance between the injured and non-injured structures on the right and left sides of the trunk and their potential interactions. The afferent impulses transmitted to the spinal cord from different receptors on the symptomatic side, mediated by interneuronal pathways, may exert crossed reflectory effects on the contralateral motor centers, a reaction that may lead to changes in motor transmission and muscle imbalances on the clinically asymptomatic side. A possible candidate that has this influence on the contralateral motor actions is the crossed disynaptic inhibitory interneuronal system [32,33]. In the available literature, there are not many neurophysiological studies that examine the consequences of BPI with results that can be compared to those presented in the current research.

In Lo and Tan’s studies [34], electrical stimulation demonstrated the existence of a multilevel motor root conduction block, which reversed after a 4-month period. Motor root conduction studies are useful diagnostic and prognostic adjuncts in the management of brachial plexopathy. The importance of the nEMG recordings obtained in this study lies in confirming the denervation process of the muscle motor units innervated from certain injured brachial plexus branches and assessing the advancement of the neurogenic changes that limit the contractile properties of single muscle motor units [35]. The predominance of injuries to the C5 and C6 cervical roots and upper trunk of the brachial plexus in our patients is similar to the results reported by Dhawan [36]. Most of the reports published by other authors provide similar etiologies and ranges of brachial plexus injury as our observations [15].

The abnormalities found in the motor neural transmission on the asymptomatic side of patients following a brachial plexus injury may have important clinical consequences. Even if they are characterized as subclinical, they may explain the poor results of BPI treatments, which sometimes appear after reconstructive surgery or conventional, long-term physiotherapeutic treatment [10,37,38]. These functional abnormalities have not been detected with standard clinical methods, but there is an opportunity to detect them when using the clinical neurophysiology methods demonstrated in this study. The symptoms of these symptomatic–asymptomatic injuries have been described in a few clinical observations [39]. This leads to the conclusion that it is necessary to add expanded neurophysiological examinations to the evaluation of the level, range, and type of brachial plexus damage seen in BPIs. A similar evaluation to the one used in this study has never been presented before; additionally, clinical studies have not excluded this possibility. Moreover, it can be concluded that a peripheral upper-trunk brachial plexus injury may mimic the typical consequences of a spinal cord injury [40]. One may argue that these symptomatic–asymptomatic changes could have been related to the patient’s positioning during these diagnostics or bias related to the precision of the measurements; however, the observed changes in neurophysiological parameters detected by our statistical analyses were not observed in the healthy volunteers. The subclinical changes demonstrated in our studies also draw attention to the asymptomatic side of BPIs, especially concerning prospective follow-up studies in this group of patients. Recording these changes may lead to modification of the treatment procedures and analysis of possible regeneration processes or the further degeneration of neural structures in clinical practice. In light of recent discoveries, our results also confirm a previous observation we made in healthy volunteers [16]. Using two kinds of stimuli (electrical versus magnetic) for the excitation of the brachial plexus’ motor components allows for a noninvasive, objective, and replicable evaluation of a patient with BPI. They can be utilized interchangeably depending on the patient’s health and ability to endure the stimulation and the requirements of the diagnostic algorithm. The currently accepted diagnostic procedures in clinical neurophysiology do not contain methodological descriptions of combining MEP tests after transvertebral cervical stimulation and at Erb’s point with ENG tests for differential diagnostics in patients with BPI [41,42,43,44]. The procedure presented in the current work is unique; electrodiagnostic abnormalities on the asymptomatic side of BPI have not yet been described.

A limitation of our study could be the number of patients examined. However, a statistical tool initially estimated that the size of our sample was sufficient to detect functional motor abnormalities on patients’ clinically symptomatic and non-symptomatic sides and demonstrate the differences in their CMAP and MEP parameters compared to those of the control group. Moreover, selecting the appropriate number of BPI patients who were a similar length of time from their moment of injury, had similar degrees of advancement in their pathology, and had a complete set of clinical and neurophysiological tests is always a challenge for researchers.

We will undertake new studies to further explore how age-related changes in the neuromuscular system may influence the results of neurophysiological examinations of patients with BPI. We established that an MEP examination of older patients (6 or 7 decades old) may provide valuable information about the functional state of their cervical roots, which may be damaged through degenerative changes in their spine. The correlation between degenerative changes in the cervical roots and the possibility of injury in the proximal part of the brachial plexus structure appears to be clinically relevant.

## 5. Conclusions

The bilateral symptoms of neurological dysfunction detected using clinical neurophysiology methods in our BPI patients show that their unilateral injury may primarily result in the functional reorganization of their spine’s internal neural connections. The electrical (ENG) and magnetic (MEP) stimuli used for the excitation of the efferent components of the brachial plexus allow for a noninvasive, objective, and repeatable evaluation of the BPI patient’s functional status and can be utilized interchangeably depending on the patient’s health to conduct therapeutic procedures or meet the requirements of a diagnostic algorithm. Future neurophysiological studies should focus on elucidating the phenomenon of clinically silent abnormalities on the asymptomatic side and the mechanisms that could contribute to their emergence in the absence of such tests.

## Figures and Tables

**Table 1:** The demographic, anthropometric, and handedness characteristics of the patients and healthy volunteers. Minimum, maximum, and mean values and standard deviations are presented.

Variable Group of Subjects	Age (Years)	Height (cm)	Weight (kg)	BMI	Handedness
Patients, N = 20					
N = 6 ♀	15–66	159–194	51.4–85.3	24.1–27.6	R = 20
N = 14 ♂	38.4 ± 13.9	174.5 ± 8.6	75.8 ± 9.4	26.1 ± 4.3	L = 0
Healthy volunteers (Control), N = 20					
N = 10 ♀	21–49	156–190	49.8–83.1	24.3–27.1	R = 19
N = 10 ♂	36.9 ± 9.8	172.5 ± 10.3	76.4 ± 11.2	25.4 ± 3.4	L = 1
*p*-value	**0.045**	0.06 NS	0.08 NS	0.07 NS	0.138 NS

Abbreviations: ♀—female; ♂—male; NS—non-significant; *p* ≤ 0.05 determines significant statistical differences, which are marked in bold.

**Table 3:** Characteristics of the brachial plexus injuries in the group of patients (N = 20).

Group of Patients	Injury Origin			Type of Injury		
		Multiorgan Trauma	Symptomatic Side	Preganglionic N = 5	Postganglionic N = 6	Mixed (Both Types of Injuries) N = 9
N = 20	Car/motorcycle Traffic accident N = 8 Iatrogenic injury N = 2 Obstetric brachial plexus injury N = 1 Other causes of damage N = 9 *	N = 6	R = 10 L = 10	C5 n = 10 C6 n = 9 C7 n = 7 C8 n = 6	All trunks n = 2 Superior trunk n = 3 Superior and middle trunks n = 6 Middle and inferior trunks n = 1 Inferior trunk n = 0 Spinal cervical nerves/anterior triangle of the neck n = 2 Whiplash syndrome n = 1	

Abbreviations: * Falls from height, shoulder girdle dislocations, arm traction, whiplash syndrome; R—right side; L—left side; N—number of the patients; n—confirmation quantity of the cervical root level of damage in patients with preganglionic, postganglionic, and mixed injuries.

**Table 4:** Comparison of results from electroneurographic examinations (ENG, CMAP) and motor-evoked potential (MEP) recordings performed in 20 patients and 20 healthy volunteers (control group).

Test Parameter	Side	Control ES CMAP	Recording Side in Patients	Patient ES CMAP	Control ES CMAP vs. Patient ES CMAP	Control MS MEP	Patient MS MEP	Control MS MEP vs. Patients MS MEP	Patient ES vs. MS	Control ES vs. MS	Control Cervical- Root-Level MSr MEP	Patient Cervical- Root-Level MSr MEP	Control vs. Patient Cervical- Root-level MSr MEP
		Min.–Max. Mean ± SD		Min.–Max. Mean ± SD	*p*-value	Min.–Max. Mean ± SD	*p*-value	Min.–Max. Mean ± SD	*p*-value	*p*-value	*p*-value	Min.–Max. Mean ± SD	*p*-value
**Axillar nerve and C5 roots stimulation/DP recording**													
Amplitude (mV)	R	10.3–18.6 14.8 ± 1.8	Symptomatic	0–21.5 7.4 ± 3.2	**0.008**	10.9–19.7 14.0 ± 2.1	0–18.0 6.8 ± 5.2	**0.008**	**0.043**	0.054	6.0–23.4 13.9 ± 4.8	0–14.0 4.2 ± 3.5	**0.008**
	L	10.2–17.6 14.3 ± 2.1	Asymptomatic	8.4–20.0 13.2 ± 3.6	0.064	9.7–18.5 13.4 ± 2.2	9.0–19.0 13.0 ± 3.3	0.053	0.069	**0.048**	6.2–22.9 14.2 ± 4.3	4.1–18.9 11.0 ± 4.0	**0.040**
*p*-value	R vs. L	0. 113	NA	**0.009**	NA	0.055	0.009	NA	NA	NA	0.063	**0.008**	NA
Latency (ms)	R	2.8–4.2 3.2 ± 0.3	Symptomatic	0–5.4 3.5 ± 1.6	0.063	2.7–3.6 3.1 ± 0.3	0–6.3 3.5 ± 1.7	**0.047**	0.241	0.188	4.0–5.4 4.8 ± 0.4	0–11.1 5.8 ± 3.1	0.052
	L	2.7–3.9 3.2 ± 0.3	Asymptomatic	2.8–4.2 3.6 ± 0.4	0.074	2.5–3.7 3.1 ± 0.3	2.7–4.1 3.6 ± 0.4	**0.041**	0.312	0.189	4.1–5.4 4.7 ± 0.4	4.3–5.9 5.0 ± 0.4	0.062
*p*-value	R vs. L	0.138	NA	0.096	NA	0.188	0.073	NA	NA	NA	0.231	0.071	NA
**Musculocutaneous nerve and C6 roots stimulation/BB recording**													
Amplitude (mV)	R	7.6–16.9 12.1 ± 2.8	Symptomatic	0–18.2 7.0 ± 5.1	**0.009**	7.2–16.7 11.7 ± 2.6	0–14 6.8 ± 4.5	**0.008**	0.061	0.057	7.9–21.5 12.6 ± 3.4	0–14.7 6.2 ± 4.6	**0.007**
	L	8.3–16.1 11.9 ± 2.2	Asymptomatic	4.3–16.2 11.2 ± 3.2	0.082	8.1–14.7 11.5 ± 2.1	6.8–15.0 11.4 ± 2.5	0.077	0.068	0.078	8.2–18.1 13.1 ± 2.9	4.5–22.9 14.4 ± 4.7	**0.046**
*p*-value	R vs. L	0.113	NA	**0.018**	NA	0.072	**0.019**	NA	NA	NA	0.082	**0.008**	NA
Latency (ms)	R	3.1–5.3 4.1 ± 0.5	Symptomatic	0–6.8 4.0 ± 1.9	0.069	3.0–4.8 3.9 ± 0.4	0–6.1 3.8 ± 1.8	0.061	0.052	0.064	4.0–6.4 5.6 ± 0.6	0–10.2 5.5 ± 2.6	0.166
	L	3.0–4.6 4.0 ± 0.4	Asymptomatic	3.4–5.6 4.3 ± 0.5	**0.048**	3.2–4.5 3.9 ± 0.4	3.4–5.0 4.2 ± 0.4	0.068	0.071	0.081	4.2–6.3 5.6 ± 0.6	4.8–7.7 5.8 ± 0.6	0.075
*p*-value	R vs. L	0.092	NA	**0.047**	NA	0.148	**0.041**	NA	NA	NA	0.288	0.062	NA
**Radial nerve and C7 roots stimulation/TB recording**													
Amplitude (µV)	R	6.6–12.7 10.4 ± 1.7	Symptomatic	0–14.9 6.0 ± 4.3	**0.014**	7.9–11.8 10.0 ± 1.2	0–15.1 6.4 ± 4.5	**0.008**	0.061	0.068	4.4–17.6 11.1 ± 3.9	0–14.1 5.7 ± 4.6	**0.007**
	L	7.3–13.0 10.4 ± 1.7	Asymptomatic	4.3–17.0 9.2 ± 3.4	**0.048**	7.1–12.3 9.8 ± 1.4	6.7–16.3 9.5 ± 2.2	0.067	0.070	0.059	5.8–18.6 10.9 ± 3.3	3.0–17.6 8.8 ± 4.0	**0.045**
*p*-value	R vs. L	0.485	NA	**0.036**	NA	0.051	**0.038**	NA	NA	NA	0.081	**0.038**	NA
Latency (ms)	R	3.0–5.1 4.1 ± 0.6	Symptomatic	0–7.2 3.8 ± 1.9	**0.034**	3.0–5.0 4.0 ± 0.6	0–6.3 3.5 ± 1.8	0.063	0.068	0.072	4.5–7.4 5.8 ± 0.8	0–7.4 5.3 ± 2.4	0.058
	L	3.0–5.8 4.1 ± 0.7	Asymptomatic	2.9–5.3 4.2 ± 0.7	**0.041**	3.0–5.5 4.0 ± 0.6	3.3–6.1 3.4 ± 0.7	0.067	**0.049**	0.092	4.5–7.1 5.9 ± 0.8	4.5–7.3 6.0 ± 0.8	0.073
*p*-value	R vs. L	0.528	NA	**0.041**	NA	0.198	0.082	NA	NA	NA	0.231	0.062	NA
**Ulnar nerve and C8 roots stimulation/ADM recording**													
Amplitude (µV)	R	1.6–14.2 5.6 ± 3.1	Symptomatic	0–12.5 5.2 ± 3.8	**0.045**	5.0–9.3 7.1 ± 1.2	0–12.0 5.2 ± 3.4	**0.038**	0.178	**0.031**	1.3–10.3 5.8 ± 2.8	0–11.2 3.4 ± 3.4	**0.042**
	L	1.0–9.7 5.4 ± 2.8	Asymptomatic	4.8–14.1 10.3 ± 3.6	**0.024**	3.6–9.0 6.2 ± 1.4	4.8–11.8 7.3 ± 2.0	**0.008**	**0.008**	**0.039**	1.0–14.1 5.4 ± 3.2	0.2–12.8 4.0 ± 3.4	**0.049**
*p*-value	R vs. L	0.074	NA	**0.035**	NA	**0.048**	**0.039**	NA	NA	NA	0.062	0.063	NA
Latency (ms)	R	10.0–13.8 12.1 ± 1.0	Symptomatic	0–17.7 11.1 ± 5.1	0.063	9.7–13.3 11.7 ± 0.9	0–17.3 11.4 ± 4.3	0.127	0.153	0.071	11.8-15.1 13.5 ± 0.9	0-19.2 13.2 ± 4.8	0.081
	L	9.9–14.4 12.1 ± 1.0	Asymptomatic	9.8–16.5 12.5 ± 1.5	0.068	9.8–13.7 11.7 ± 1.0	10.6–16.4 12.4 ± 1.5	**0.040**	0.211	0.073	12.0–15.1 13.6 ± 0.9	12.3–18.7 14.6 ± 1.7	0.056
*p*-value	R vs. L	0.485	NA	**0.043**	NA	0.142	0.063	NA	NA	NA	0.119	0.052	NA

Abbreviations: ES—electrical stimulation at Erb’s point, MS—magnetic stimulation at Erb’s point; MSr—magnetic stimulation of the cervical roots levels; CMAP—compound muscle action potential; MEP—motor-evoked potential; DP—deltoid posterior muscle; BB—biceps brachii muscle; TB—triceps brachii muscle; ADM—abductor digiti minimi muscle; C5–C8—cervical root levels; NA—non-applicable; NS—non-significant; *p* ≤ 0.05 determines significant statistical differences marked in bold.

**Table 5:** Results of sensory nerve conduction studies (SNCSs) and needle electromyography studies (nEMG) conducted on patients (N = 20).

Test	Score/N = 20
SNCS	
Median nerve	
D1	0/2 1/8 2/10
D2	0/2 1/6 2/12
D3	0/2 1/5 2/13
Radial nerve (snuff box)	0/3 1/5 2/12
Ulnar nerve D5	0/2 1/5 2/13
nEMG	
DP	DD 0/12 1/8; MUAP 0/5 1/11 2/4
BB	DD 0/11 1/9; MUAP 0/3 1/13 2/4
TB	DD 0/13 1/7; MUAP 0/4 1/9 2/7
FDI	DD 0/13 1/7; MUAP 0/2 1/10 2/8

Abbreviation: D1–D5—sensory examination of first to fifth digits; DP—deltoid posterior muscle; BB—biceps brachii muscle; TB—triceps brachii muscle; FDI—first dorsal interosseous muscle; SNCS grade: 0—lack of evoked potential, 1—pathological parameters of evoked potential, 2—normal parameters; nEMG grade: DD—denervation discharges, 0—lack of denervation potentials, 1—denervation potentials recorded; MUAP—motor unit action potentials: 0—lack of voluntary muscle activity, 1—neurogenic MUAPs, 2—normal parameters of MUAPs.

## Data Availability

All the data generated or analyzed during this study are included in the published article.
